# Prognostic Value of the Systemic Immuno-Inflammation Index (SII) in the Formation of Seroma and Hematoma Following Thyroidectomy

**DOI:** 10.1055/s-0045-1810078

**Published:** 2026-03-03

**Authors:** Gokhan Toptas, Hande Arslan, Sumeyra Doluoglu, Sevket Aksoy, Latif Akan, Esma Altan, Omer Bayir, Guleser Saylam, Mehmet Hakan Korkmaz

**Affiliations:** 1 Department of Otolaryngology, Head and Neck Surgery, Ministry of Health Ankara Etlik City Hospital, Ankara, Turkey; 2 Department of Otolaryngology, Head and Neck Surgery, Ministry of Health Samsun Education and Research Hospital, Ilkadim, Samsun, Turkey; 3 Department of Otolaryngology, Head and Neck Surgery, Lokman Hekim Universitesi, Ankara, Turkey; 4 Department of Otolaryngology, Head and Neck, Self-Employement Special Medical Clinic, Ankara, Turkey

**Keywords:** hematoma, seroma, SII, Thyroidectomy

## Abstract

**Objective:**

This study aims to evaluate the prognostic value of the Systemic Immuno-Inflammation Index (SII) for predicting the development of seroma and hematoma after thyroidectomy.

**Methods:**

A retrospective analysis was conducted on 127 patients who underwent thyroidectomy between January 2017 and May 2022. Patients were categorized into two groups: those who developed seroma or hematoma (study group) and those who did not (control group). Preoperative SII scores were calculated using routine blood parameters.

**Results:**

Of the 127 patients included, 29 (22.8%) developed seroma or hematoma postoperatively. The mean preoperative SII score was significantly higher in the study group compared with the control group (750 ± 86.3 versus 591.5 ± 32.8; *p* = 0.04). ROC analysis determined an optimal SII cutoff value of 535, with a sensitivity of 76% and specificity of 53% for predicting these complications.

**Conclusion:**

The preoperative SII score may serve as a useful predictor for the development of seroma and hematoma following thyroidectomy. Further studies with larger sample sizes and prospective designs are needed to validate these findings and refine the clinical applicability of SII in predicting postoperative complications in thyroid cancer patients.

## Introduction

The most common endocrine cancer is thyroid malignancies. In 2020, there were 586,000 new cases of thyroid cancer worldwide, ranking ninth among tumor types. 1 In recent years, there has been a significant increase in the incidence of thyroid cancer, which has garnered attention and resulted in a rise in surgical indications. Studies have shown that this is particularly associated with the increase in small differentiated papillary carcinomas of the thyroid. 2 3 The increase is believed to be attributed to various factors coming together, including environmental influences, an increase in radiation exposure, advancements in radiodiagnosis and cytopathologic techniques, as well as the development and accessibility of chemical and molecular tests.

It is beyond question that surgery plays an essential role in the treatment of thyroid cancers. While thyroidectomy is generally considered a safe surgical procedure, it can potentially give rise to postoperative complications that are of clinical significance. Recognized complications of thyroidectomy encompass hypoparathyroidism, laryngeal nerves injury, hematoma and seroma. 4 Although rare, the occurrence of a hematoma following thyroidectomy is a severe complication that has the potential to be life-threatening, with reported incidence ranging from 0.7% to 4.7%. 5 6 7 Seroma is seen in around 1–2%., and the underlying mechanisms of seroma remain unclear. 8 9 In previous studies, various factors have been identified as associated with an increased risk of complications following thyroidectomy. These factors include patient characteristics such as sex, the use of drains, the creation of subplatysmal flaps, central neck dissection, and specific thyroid diseases like Graves' Disease. Additionally, the surgeon's experience and the hospital's operative volume have also been linked to higher rates of postoperative hematoma and seroma. 4 10 11 12

The patient's baseline blood parameters and the status of the systemic immune response before surgery can also be regarded as potential contributors to susceptibility to complications. Recently, several leukocyte-based inflammatory markers, such as the Systemic Immune Inflammation Index (SII), platelet-to-lymphocyte ratio (PLR), lymphocyte-to-monocyte ratio (LMR), and neutrophil-to-lymphocyte ratio (NLR), have emerged as potential predictors for the diagnosis and prognosis of diverse diseases. SII, initially developed in 2014, is deemed to possess greater predictive capabilities in comparison to NLR and PLR. 13 Furthermore, SII is believed to provide insights into the equilibrium between the host's immune response and inflammatory conditions. 14

The aim of this study is to investigate the predictability of SII for the development of seroma and/or hematoma in the postoperative period. Thus, by identifying patients who are at a higher risk of developing seroma and/or hematoma during follow-ups after thyroidectomy, we aim to predict which patients require closer monitoring during the postoperative follow-up period.

## Methods

This study was conducted retrospectively in the otolaryngology clinic between January 2017 and May 2022, with approval from the Clinical Research Ethics Committee under decision number 135/03.

The study included patients over the age of 18 who underwent total thyroidectomy with ipsilaterally or bilaterally central neck dissection due to papillary thyroid carcinoma. Patients who had previously received radiotherapy or chemotherapy for any reason, those who had surgery in the head and neck region, those with chronic diseases (such as diabetes mellitus, connective tissue disease, heart-kidney-liver disease, etc.), those with nodules larger than 7 cm on ultrasound, and those with retrosternal goiter were excluded from the study. All patients underwent a thorough physical examination, a history review, and an indirect laryngoscopy to evaluate the mobility of the vocal cords.

The demographic data of the patients were reviewed within the parameters of the study's data, including age, sex, thyroid ultrasonography data, pathological subtypes of papillary carcinoma, and whether central dissection was performed unilaterally or bilaterally. Complete blood count, blood glucose level, renal function tests, liver function tests, coagulation profile, thyroid function tests (T3, T4 and TSH), and thyroid antibodies (in selected instances) were recorded. Blood samples for complete blood count parameters were analyzed with hematology analyzer (CELL-DYN Ruby Hematology System, Illinois, USA). Blood samples were taken routinly from all participants after at least 8-hour overnight fasting within 3 days before the opeartion for preoperative evaluation by both surgeons and anesthesiologists. None of the patients were on their menstrual period when taking a blood sample. The systemic immune inflammation index (SII) is a blood test based inflammatory marker that is determined as follows: SII = neutrophil count × platelet count/lymphocyte count.

Of the 127 patients included in the study, 29 developed seroma-hematoma and were designated as the study group. The remaining 98 patients, who did not develop seroma-hematoma, were designated as the control group.

### Operative Procedures

All surgeries were performed by experienced surgeons or under their primary supervision, using a Kocher incision. All patients underwent surgery under general anesthesia and were intubated with tubes containing intraoperative neuromonitoring-compatible electrodes, and all patients underwent open total thyroidectomy with CLND. The skin and subcutaneous tissues were separated and then, the midline of the strap muscle was divided to expose the thyroid gland. Dissection of the total thyroid and central lymph node was performed, and the parathyroid glands and RLN were preserved. During central lymph node dissection, all lymph nodes (level VI) located in the region bordered by the carotid sheath laterally, the trachea medially, the hyoid bone superiorly, and the suprasternal notch inferiorly removed. The wound was sutured, but no drainage tube was inserted. All operations used vessel sealing devices and bipolar coagulation forces to stop bleeding.

All patients who developed hematoma were taken back to surgery, and bleeding was controlled using suture ligation, vessel sealing device and bipolar coagulation forceps. Patients who developed seroma were managed more conservatively. Initially, daily follow-up was conducted, and seromas were expected to resolve on their own through repeated aspirations.

### Statistical Analysis

The analysis of the results was performed using the IBM SPSS Statistics Version 21.0 software for Windows (Armonk, NY). Data was tested for normal distribution using the Kolmogorov-Smirnov test. Data were expressed as mean ± standard deviation (SD) for data with normal distribution, and median (minimum–maximum) for data without normal distribution. Student *t* -test (for two groups) was used for independent samples to analyze SII scores, and Red-cell Distribution Width. The Mann-Whitney U test was used to compare groups without normal distribution. The Chi-square test was performed for categorical variables. Statistical significance was defined as *p* < 0.05.

## Results

Four of the 131 patients included in the study were excluded due to wound infection. The remaining 127 patients were included in the study. The mean age of the patients was 45.6 ± 2.2 years, 110 (86.6%) were female and 17 (13.4%) were male. The mean age in the study group was 48.2 ± 1.6 and in the control group it was 44.8 ± 3.2. In the study group, 19 (65.5%) of 29 patients developed seroma and 10 (34.5%) developed hematoma. There was no significant difference between the study group and control group in terms of gender and age (p: 0.94 and p:0.23, respectively).

In the study group 8 (27.6%) patients had ipsilaterally, 4 (13.8%) patients had contralaterally, and 17 (58.6%) patients had not any lymph node with suspected metastasis. In the control group 34 (34.7%) patients had ipsilaterally, 23 (23.5%) patients had contralaterally, and 41 (41.8%) patients did not have any lymph node with suspected metastasis. There was no significant difference between the study group and control group in presence of lymph node with suspected metastasis (p: 0.26).

In the study group central neck dissection was performed in 12 (21.4%) patients ipsilaterally and in 17 (58.6%) patients bilaterally. In the control group, the central neck dissection was performed in 44 (44.9%) patients ipsilaterally and in 54 (55.1%) patients bilaterally. There was no relation between central neck dissection and postoperative seroma/hematoma formation (p: 0.74). In the preoperative SII scores in the study, the control group had a mean score of 591.5 ± 32.8 (mean ± SD), while the study group had a mean score of 750 ± 86.3 (mean ± SD). There was a statistically significant difference in the preoperative SII scores between the study group and the control group (p: 0.04)

The postoperative pathology results showed that 20 (69%) patients had classic papillary thyroid carcinoma, 8 (27.6%) patients had follicular variant papillary thyroid carcinoma, and 1 (3.4%) patient had oncocytic variant of papillary thyroid carcinoma in study group. In control group 71 (72.4%) patients had classic papillary thyroid carcinoma, 24 (24.5%) patients had follicular variants of papillary thyroid carcinoma, and 3 (3.1%) patients had oncocytic variant of papillary thyroid carcinoma ( Table 1 ).

**Table 1:** Characteristic and demographic data of patients (Control /Study Group)

	Control Group (N:127)	Study Group (N:29)	*p*
Age	44.8 ± 3.2	48.2 ± 1.6	0.23
Gender			0.94
- Female - Male	85 13	25 4	
Seroma/Hematoma	–	19/10	
Neck dissectıon			0.74
- Ipsilaterally - Bilaterally	44 (%44,9) 54 (%55,1)	12(%21,4) 17(%58,6)	
Lymph node			0.26
- Ipsilaterally - Contralaterally	34 (%34.7) 23 (%23,5)	8(%27,6) 4(%13,8)	
Pathology			0.96
- Classic PTC - Follicular variant of PTC - Oncocytic variant of PTC	71 (%72,4) 24 (%24,5) 3 (%3,1)	20 (%69) 8 (%27,6) 1 (%3,4)	

Abbreviation: PTC, papillary thyroid carcinoma.

There was no relation between postoperative pathology results and postoperative seroma/hematoma formation (p: 0.96).

Preoperative hematologic findings with normal distribution were shown in Table 2 . Preoperative hematologic findings with normal distribution were shown in Table 3 .

**Table 2:** Preoperative hematologic parameters of patients

	The study group (median (min-max)) (n: 29)	The control group (median (min-max)) (n: 98)	*p* value
Hemoglobin (g/dL)	13.2 (11.6–17.1)	13.5 (11.1–17)	0.32
Platelet (10 ^3^ /μL)	294 (155–460)	260 (140–431)	0.15
Neutrophil count (10 ^3^ /μL)	4.6 (2.3–9.59)	4.3 (2.5–8.7)	0.95
Lymphocyte count (10 ^3^ /μL)	1.9 (0.9–4.7)	2.2 (1.2–4.8)	0.04

Mann-Whitney U test were used to compare groups.

**Table 3:** Preoperative Red-cell Distribution Width and SII scores of patients

	The study group (mean ± SD) (n: 29)	The control group (mean ± SD) (n: 98)	*p* value
Red-cell Distribution Width (RDW)	13.9 ± 0.3	14 ± 0.2	0.75
SII	750 ± 86.3	591.5 ± 32.8	0.04

Student *t* -test was used to compare groups.

**Fig. 1:**
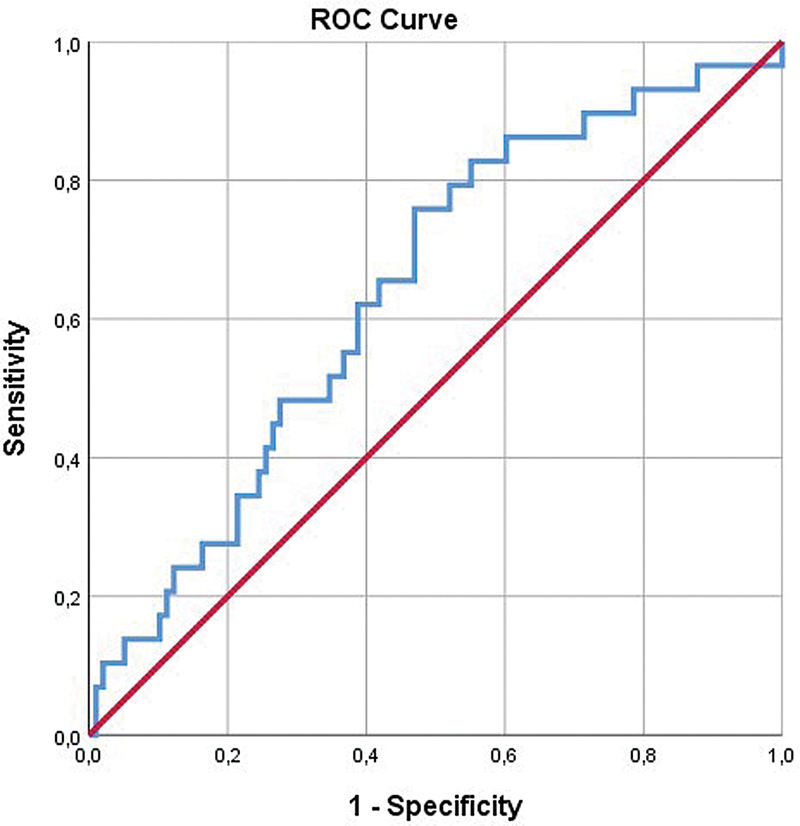
ROC analysis for systemic immune-inflammation index (SII).

Based on receiver operating characteristic (ROC) analysis, the cut-off level for SII that was associated with the postoperative seroma/hemotoma was 535 mg/L. The area under the ROC curve was 0.640, with a sensitivity of 76% and specificity of 53% ( Fig. 1 ). The probability of seroma-hematoma was found to be lower in patients with SII ≤ 535 mg/L compared with patients with SII> 535 mg/L.

There was no relation between SII scores and postoperative pathology results of all patients participating in the study. The SII score was 656.24 ± 37.8 in patients with classic papillary thyroid carcinoma, 569.31 ± 69.5 in patients with follicular variant of papillary thyroid carcinoma and 444.6 ± 52.8 in patients with oncocytic variant of papillary thyroid carcinoma (p:0.31).

## Discussion

In recent years, there has been a notable increase in the incidence of thyroid cancer, which has garnered significant attention and led to a rise in the number of surgical indications. When determining surgical indications, patient selection and identifying individuals at higher risk of developing complications can assist the surgeon during the preoperative period. In our study, we investigated whether the systemic immune-inflammation index (SII) could help predict the likelihood of seroma or hematoma development before thyroidectomy.

Thyroidectomy can lead to several postoperative complications, including hypoparathyroidism, infections, and seromas, which increase morbidity, prolong recovery, and extend hospital stays. 15 Due to the high vascular supply of the thyroid gland, intraoperative heedful hemostasis is essential to prevent major complications especially hematoma. A study in Italy, which included 2559 patients who underwent thyroid surgery, found the incidence of hematoma to be 1.36%. 16 In another multicenter study involving 8839 patients, the incidence of hematoma after thyroid surgery was determined to be 3.15%. 17 In our study, hematoma developed in 10 (7.8%) of the 127 patients, which is consistent with the literature. Additionally, in our study, fibrin sealant application was not used; instead, all operations employed vessel sealing devices and bipolar coagulation forceps to control bleeding.

The incidence of seroma following thyroidectomy ranges from 1.3% to 14%. Although not life-threatening, seromas are associated with patient discomfort and morbidity, including pain, regional swelling, surgical site infection, prolonged hospital stay, and increased healthcare costs. 10 11 The etiology and pathophysiology of seromas are not fully understood. Several factors, such as the oozing of small vessels, cavity creation from tissue removal, lymphatic vessel lesions, and inflammatory responses, have been reported to influence seroma formation. 18 However, the mechanism for preventing seroma formation after thyroidectomy remains unclear. 19 The routine use of drains after thyroid surgery is controversial because they may cause an inflammatory response, infection, and discomfort. 20 In addition, the use of a drain has a very limited effect in preventing hematoma. 21 The placement of drain is more likely to help the flaps to settle. In our study, no drains were used, and patients with drains were specifically excluded to maintain a homogeneous patient group.

Previous studies have shown that thyroid histology, whether benign or malignant, may influence postoperative bleeding. 22 23 Our results align with recent meta-analyses that found no association between pathological findings and the risk of hematoma formation. 24 25 We observed no association between postoperative pathology findings and the occurrence of postoperative seroma or hematoma ( *p* = 0.96). This may be because patients with early-stage tumors were selected to create a homogeneous group.

It is postulated that the SII may offer insights into the dynamic equilibrium between the host's immune response and inflammatory conditions. The SII index is a novel indicator reflecting the balance of the inflammatory response and the immune status of the host in a way that was previously not possible. Although the index's utility was first described by Hu et al to predict hepatocellular carcinoma recurrence, its value has been studied in various other diseases, including sepsis and dental infections. 26 27 28 However, its potential in predicting thyroid seroma and hematoma had not been previously evaluated. This area is just beginning to be explored and has the potential to revolutionize disease diagnosis and treatment.

No definitive cutoff value for the SII has been established. In patients with deep neck infections, a SII cutoff value of 2975 was found. 29 In obstructive sleep apnea patients, a cutoff value of 290 was determined. 30 In our study, the SII cutoff value for patients with seroma and hematoma after thyroidectomy was found to be 535 mg/L, with 76% sensitivity and 53% specificity.

The study's limitations include its retrospective nature and the combined evaluation of patients with seroma and hematoma. However, cases considered risk factors for these complications, such as revision thyroidectomy, retrosternal thyroidectomy, and parathyroid surgery combined with thyroidectomy, were excluded to create a homogeneous patient population. Future research should consider increasing the number of patients and evaluating these groups separately.

The preoperative SII scores of the study group and the control group were statistically significantly different. But our study found no relationship between central neck dissection and the formation of postoperative seroma or hematoma. Additionally, we observed no association between postoperative pathology results and the occurrence of postoperative seroma or hematoma.

## Conclusion

In conclusion, while the SII may show promise as a predictor for complications such as seroma and hematoma following thyroidectomy further research with larger patient populations and prospective studies is needed to validate these findings and refine the clinical utility of the SII in this context.
